# Case-study of a user-driven prosthetic arm design: bionic hand versus customized body-powered technology in a highly demanding work environment

**DOI:** 10.1186/s12984-017-0340-0

**Published:** 2018-01-03

**Authors:** Wolf Schweitzer, Michael J. Thali, David Egger

**Affiliations:** 1 Zurich Institute of Forensic Medicine, University of Zurich, Winterthurerstrasse 190, Zürich, Switzerland; 2 Balgrist Tec, Forchstrasse 340, Zürich, Switzerland

**Keywords:** Prosthesis design, Artificial limbs, Artificial arm, Body-powered prosthetic arm, Myoelectric prosthetic arm, User-driven design

## Abstract

**Background:**

Prosthetic arm research predominantly focuses on “bionic” but not body-powered arms. However, any research orientation along user needs requires sufficiently precise workplace specifications and sufficiently hard testing. Forensic medicine is a demanding environment, also physically, also for non-disabled people, on several dimensions (e.g., distances, weights, size, temperature, time).

**Methods:**

As unilateral below elbow amputee user, the first author is in a unique position to provide direct comparison of a “bionic” myoelectric iLimb Revolution (Touch Bionics) and a customized body-powered arm which contains a number of new developments initiated or developed by the user: (1) quick lock steel wrist unit; (2) cable mount modification; (3) cast shape modeled shoulder anchor; (4) suspension with a soft double layer liner (Ohio Willowwood) and tube gauze (Molnlycke) combination. The iLimb is mounted on an epoxy socket; a lanyard fixed liner (Ohio Willowwood) contains magnetic electrodes (Liberating Technologies). An on the job usage of five years was supplemented with dedicated and focused intensive two-week use tests at work for both systems.

**Results:**

The side-by-side comparison showed that the customized body-powered arm provides reliable, comfortable, effective, powerful as well as subtle service with minimal maintenance; most notably, grip reliability, grip force regulation, grip performance, center of balance, component wear down, sweat/temperature independence and skin state are good whereas the iLimb system exhibited a number of relevant serious constraints.

**Conclusions:**

Research and development of functional prostheses may want to focus on body-powered technology as it already performs on manually demanding and heavy jobs whereas eliminating myoelectric technology’s constraints seems out of reach. Relevant testing could be developed to help expediting this. This is relevant as Swiss disability insurance specifically supports prostheses that enable actual work integration. Myoelectric and cosmetic arm improvement may benefit from a less forgiving focus on perfecting anthropomorphic appearance.

## Background

Work specific tasks [1] invariably define specific functional requirement profiles for workers (including prosthetic arms) [2]. Once a personal preference has expressed itself in the form of particular work choice, and once a person has acquired extensive experience and skills in a particular field, particular technical necessities often follow with little further options [3]. Then, various prosthetic solutions may be thought to be competing for better performance when in fact, the choice-dependent hard requirements for a viable prosthetic solution have already set the stage. Primarily, any competition seems to boil down to body-powered versus myoelectric technology [4]. Within body-powered control systems, voluntary opening (VO) and voluntary closing (VC) devices offer different profiles [5].

Assessment of current state and developments of prosthetic arms presented here has one particular aim. That aim is to enable the first listed author of this paper^1^ to keep working, at the front, within one of the most modern forensic pathology institutes and projects [6]. Our focus, therefore, is the occupational rehabilitation for one particular profession. Since 2008, the first author develops, tests and refines solution oriented prosthetic arm components (as detailed in this case study)^2^.

With a generic task choice based on ADL (activities of daily living), the CYBATHLON 2016 [7] had provided a competitive demonstration of prosthetic arms in October 2016 in Zürich, Switzerland. There, competitors wearing prosthetic arms attempted both fast and precise manipulations performing light activities. A televised public arena setting [8] provided for a certain degree of intensity and stress. The winner wore a body-powered arm; the myoelectric arm users filled the remaining ranks.

Intensity in physically demanding tasks, such as discussed in this paper, will be a lot greater along more than one dimension. Dimensions include wider ambient temperature range, longer duration of work, heavy sweating and far larger pull or push weights. There is also a more existential aspect of manipulation content, i.e., an accidental drop of an expensive camera is penalized more unforgivingly than not winning a medal.

In this paper, we will employ the term “physically demanding work” (PDW) to denote physically intense, repetitive, hazardous, demanding, unforgiving, critical and otherwise extensively bi-“manual” work. It demands undivided attention, it does not provide extra time to troubleshoot the prosthesis, and it requires full reliability for pull, push, lift or grip manipulations [9].

### What is the current requirement for prosthetic arm technology?

Individual job assignments determine tasks the arm amputee has to solve, and that their prosthesis must address. The majority of acquired adult major arm amputations are traumatic unilateral below elbow amputations (UBEA) (77% in [10]), with a predominance of blue collar^3^ workers. In that community, occupation-specific manual tasks tend to be hazardous, repetitive, strenuous and hard [1, 11, 12]. These tasks then also should be at the core of rehabilitation; if they are not, unemployment and a need for re-schooling risk to follow [3].

The aspect of PDW is not likely to go away. Even in the light of ongoing automatization and technological advance, athletic, physical and manual skill requirements remain relevant while the demands for an extreme degree of fine manual skill is not excessive [13, 14]. Priorities are high reliability concerning device integrity, reliable control under physical strain even with sweaty skin [15] and reduction of overuse or asymmetry problems under full load and over time.

Evaluating the impact of wearing a prosthetic arm on overuse and asymmetry may require load and hazard stratification. There are studies that discuss overuse and asymmetry consequences [16, 17], also in the context of wearing a prosthetic arm [18–22]. They do not address the fact that for very intense work, it may make a significant difference for that individual whether a prosthesis is worn that actually supports intense work, and whether the individual trains to keep fit for that job.

One particularly exposed group of workers are farmers. They report a high degree of exposure with wide ambient temperature ranges, corrosive or damaging liquids, particles, biological and chemical contaminants as well as extensive wear and tear of general work. The exposure goes so far beyond the usual prosthetic technician’s scope that the authors of one farmer focused study called it ‘extraordinary’ [23].

Activities of daily living (ADL) such as putting on overalls, folding clothes, reading a newspaper, loading a vehicle with equipment, drinking water from a cup, showering or preparing a meal do require some degree of manual dexterity [24]. The same manipulations that make up the ADL inventory find themselves in considerably more unforgiving industrial variations across hazardous occupations, where they are performed with high frequency, with high load, under heat exposure and with far less tolerance as to errors [25]. A UBEA may well be able to provide full-time PDW by, e.g., repairing bicycles, working in a gastronomical kitchen, or, providing biology laboratory work. That individual then will be delivering adequate “motor performance” in a demanding environment. That person is not likely to experience any functional deficiencies concerning the more limited scope of “motor capabilities” required by ADL [26].

This is illustrated by the CYBATHLON 2016 Arm Prosthesis Race that was won by a 67-year old pilot equipped with a body-powered TRS Grip 5 Evolution Prehensor. That is a light build of the TRS Adult Prehensor, which features a metal frame. These VC devices allow for any grip between very subtle careful handling e.g. of a light bulb or an egg [27], up to regular and long term usage of shovels or picks or handling of heavy weights. Body-powered VC control transmits adequate proprioception [28], particularly as to grip strength, even under heavy sweat. At the CYBATHLON 2016, a light version of a body-powered prosthetic arm system fully geared towards PDW requirements made ADL-optimized systems pale within their own application domain.

Current prosthetic arms are weak especially in supporting industrial work such as machining, processing, and construction. After suffering an arm amputation, the category of workers formerly employed for heavy work is related to the highest fraction of industrial workers changing jobs [3]. However, re-schooling is both costly and risky as it can have serious complications (such as depression, increased divorce rate or increased mortality [29, 30]). Depression is already prevalent among arm amputees [31] and it adds to complicating prosthetic rehabilitation [32].

By law, insurances usually are restricted to financing cost-effective prostheses. Cost-relevant aspects are both the ability to return to work, where applicable also heavy work, and long term health in context of the prosthetic costs^4^.

The current requirement for prosthetic arm technology is to work particularly well under realistic conditions where bi-manual work is mandatory. These typically comprise high exposure and low failure tolerance.

### What is the current acceptance for prosthetic arm technology under these requirements?

In the best case, a conventional prosthetic arm offers marginal functional improvements [33]. Subgroup rejections are reported to be as high as 59% (for amputations close to the wrist [34]) or 75% (for myoelectric prostheses [35]). As opposed to reported figures, realistic rates for rejection and non-usage have been estimated to be even higher due to absent contact between the clinic community and non-users [36]. A non-response following unsuccessful purchase as entity is generally kept proprietary and not released in the public domain, whereas 40% of dissatisfied customers were estimated do nothing about it and only 5% escalated their complaint to management [37]. The underlying mechanism likely is a significant degree of mutual disengagement [38, 39]. It therefore can be assumed that most arm amputees, particularly those that do not submit to heavy work, reject prosthetic arms.

The situation will be different for workers. Generally, across various physical activity levels of jobs, over half of the employed workers with amputations identified negative repercussions of their amputation, and one quarter employed at the time of the study had experienced unemployment lasting over six months since their amputation [3]. The lowest percentages of workers returned to “heavy” or “very heavy” work while the trend was that 75% of employed amputees returned to jobs that were less heavy but required greater intellectual ability [3].

A body-powered split hook or prehensor [40] dominates in successfully supplementing most users that are involved in PDW [3, 41–43], and not a myoelectric arm. If one focuses on body-powered technology and on adult below elbow amputees that are in the work force, one study [44] reported 10/10 of below elbow dominant arm amputees and 17/19 of all below elbow amputees having become users during a study period of 7 years. In another study, body-powered arms supported a majority of workers also delivering heavy variable work in excess of 8 hours per day [45], while work load as well as popularity was considerably lower for myoelectric or passive arms. This has not changed since the invention of myoelectric prostheses [44, 46].

## Forensic medicine as a work environment

This section describes technical aspects of forensic medicine field work, office and laboratory work. It is physically and technically demanding. Requirements exceed the usual scope of amputee rehabilitation [47]^5^.

**Fig. 1:**
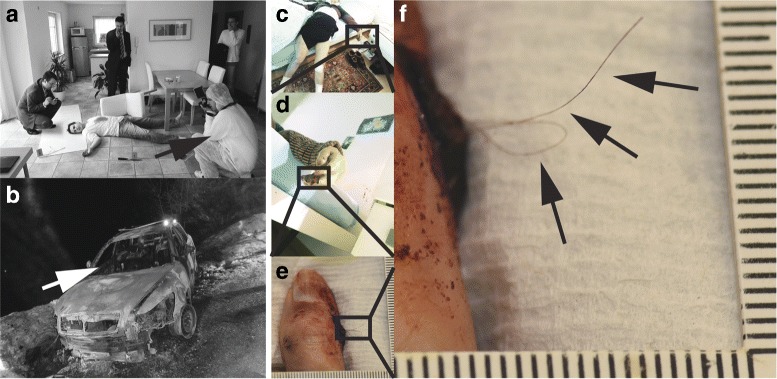
On location – Death scenes that warrant a board certified senior forensic pathologist to travel on location and perform a full body investigation with forensic scientists and photography usually are “extraordinarily extraordinary”. Indoors or outdoors work [**a**: simulated/staged teaching death scene mock-up showing protective gear (arrow) – the body will be fully undressed, without cutting clothes, and then turned over and back while obtaining a detailed body surface inspection; **b**: outdoors death scene with burn victim on passenger seat (arrow) in a -15 deg C winter night with ice and snow covered roads] usually is problematic on several levels; at this particular death scene with the burnt car, several specialists repeatedly fell to the ground due to extremely slippery and steep ground. Undressing and examining a body from all angles (**c**: deep hand / finger injury, details in D through F) requires careful preservation of losely attached evidence so that even an attacker’s hair remains in place (**c**, **d**: dressed body; **e**, **f**: undressed). Attacker was a cat in this instance

Routine death scene investigations involve handling, maintenance, cleaning and transport of equipment, They include carrying equipment also to remote locations. There is handling, undressing, turning and moving of bodies across the human weight range. Work is performed personally by the forensic pathologist, with a specific goal to not place new injuries on the body (Fig. 1). Work also entails bi-manual instrument handling and evidence collection (forceps, scalpel, dissection, syringes, swabs), One may have to manipulate fragile, putrefied, slippery or severely injured bodies and disjunct body parts. External factors may be wide ambient temperature ranges, fluid or gaseous biohazards, 24-h on-call work and wearing full body protective overalls. One requires a 24/7 fitness to drive at all weathers. The job entails associated heavy sweating [48]. In our institute, physical requirements have increased slightly over the years for the forensic pathologist as both average body weight [49] and deployment rates have gone up.

Potentially prosthesis-critical tasks for forensic field work, laboratory work and office work are summarized in Tables 1 and 2. The job description knows no specific requirement regarding which upper limb performs which work step. There is no explicit requirement or regulation as to wearing a prosthetic arm or using a particular terminal device. However, control and suspension issues as well as grip-specific differences result in different success rates across terminal devices (see Tables 1 and 2). Lack of bi-manual support is problematic for large weights and for some technical examination and handling steps.

**Table 1:** Prosthetic suspension, control and overall prosthesis related observations

Task/issue	TBI (iLimb)	CBPA (body-powered)
Carry items for more than 8m distance^b^.	Frequent drops due to postural interference [90, 91, 140]^ab^.	No problem.
Pulling or lifting bodies (typically 60–90 kg).	Excessive pull displaced or detached skin-electrode contact [92] and strained stump skin mechanically [72]. Socket (suspension) fit was not sufficiently robust for heavy pulling or holding^a^.	No problem.
Sweat issues^b^.	Electrode control loss after 10 min with shortest examinations of 30 min [84, 85]^a^. Electrode rash with sweat exposure, slow healing over ∼ 6 weeks [98–100]^a^.	No problem other than having to pour out sweat occasionally.
Typing^b^ [216].	Shoulder and elbow pain due to (a) distal weight / center of COG [87] and (b) compensatory posture [178] required to fit awkward hand shape into typing position. Skin abrasions and blisters on stump skin due to friction occured already over a few hours [72]^b^.	No problem. Weight and design of split hook, prehensor and wrist optimal for highly repetitive hard push operations. Ideal posture with Hosmer model 5 series hooks.
Meetings, presentations^b^.	The prosthesis attracted unwarranted and irritated attention, also by being unreliable, which in part was seen a consequence of limb positioning effect [90, 91], myoelectrode dysfunction [84, 85] and grip problems (see Table 2).	The CBPA’s wrist unit allowed for rapid swaps of the terminal device (split hook) against a prosthetic hand. such as the Becker hand. As this device worked flawlessly with regard to grip reliability and no noise, it did not distract others nearly as much.
Overall reliability.	Battery [95], sweat, socket fit, electrode function (“bad hand day”) [84, 85], software issues and hand grip control issues lead to a rating as insufficient reliability for the job and tasks evaluated here.	Occasional repair necessary after wear down of cable or supporting structures, with a frequency of about once every 9 to 12 months.
Cost for operating or running the device^b^.	Each glove around 300-700 USD, lasting up to 10 min even under light work conditions. Device at 80,000 USD. For a 3 year period of use with 12 weeks of on-call work, 10 hours per day with 7 days a week, assuming that a glove withstands 3 hours of actual work usage (which it does not), an hourly hardware cost of 198 USD/ hour is obtained.	No glove or weardown issues; split hook claws can be covered with silicone tubing if required (a few cents per fitting). Prosthetic arm at 6,000 USD, custom shoulder anchor at 3,000 USD, custom wrist at 1,500 USD, prosthetic split hook or prehensor at 400 to 1,200 USD. Becker hand 650 USD. For the same 3 year period of use, hourly hardware cost of about 5 USD results (about 2% of TBI).

^a^Not acceptable / not negotiable in work environment

^b^Rated just as good or better without prosthesis

**Table 2:** Terminal device related observations

Task	TBI (iLimb)	CBPA (body-powered) - Hosmer hook 5XA	CBPA (body-powered) - TRS adult prehensor	CBPA (body-powered) - Becker hand
Loading car, carrying items for more than 8m distance.	Useful grip for cut out box handles, not useful for recessed grip grooves. Limb positioning effect [90, 91] with sudden drops^a^.	No problem. Great balance for posture^c^.	No problem. Requires focus to keep cable tense (VC paradigm).	No problem. Great balance for posture.
Taking notes holding notepad in the field	Feasible but not ideal. Clumsy grip types with unforeseeable force vectors^b^.	No problem.^c^	No problem but requiring focus to keep cable tense (VC paradigm)^b^.	No problem.
Undressing body	Grip by far too weak^b^.	Good support, finding right angle for best grip requires attention^b^.	Perfect support, top application domain for VC control paradigm.^c^	Perfect support due to shape and built-in grip lock.
Turning or transporting body (Fig. 11a-h)	Grip by far too weak. Suspension started to come off^b^.	Good support, but grip forces sometimes not large enough^b^.	Perfect support, top application domain for VC control paradigm.^c^	Good support, but grip forces sometimes not large enough^b^.
Lab work (Fig. 11i-k)	Grip too weak. Limb positioning effect [90, 91] with sudden drops^a^. Unforeseeable grip force vectors [93]^b^.	Good support, but grip forces sometimes not large enough.	Perfect support, top application domain for the VC control paradigm^c^.	Good support, but grip forces sometimes not large enough.
Cleaning, disinfecting terminal device.	Costly thin cosmetic glove not to be replaced by user; no work rubber glove to be worn according to manufacturer [89].	Metal split hooks easy to swap or disinfect. Wearing thick rubber glove over hook device not technically difficult and not prohibited by manufacturer.	Wearing rubber glove fingers to TRS prehensor allows for easy disinfection; not prohibited by manufacturer specifications.	Becker hand fits humanly used rubber protection gloves, that can be worn as they both fit well and are not prohibited by manufacturer specifications.
Typing	COG too far in front, device too heavy. Device shape and weight cause shoulder pain. Suspension results in severe friction problems on stump skin after long typing^ab^.	Perfect.^c^	Good, but Hosmer 5XA has the better angle^b^.	COG too far in front, and Hosmer 5XA has the better angle^b^.
Meetings, presentations	Grip problems as the hand does not handle large pens with difficult to tear of caps well. Loud source of distraction [96, 97]^b^.	Handles paperwork and pens well.	Handles paperwork and pens well.	Perfect, least amount of distraction with grip suited well to typical tasks^c^.
Soft covers for terminal device	Gloves very thin to avoid impeding hand mechanism. Perforating damage [87], as soon as 10 min into light work. Approved gloves costly, only sold by manufacturer.	Silicone tubing affordable, easy to mount, enhance form closure. Under full work load, replace after 1–2 weeks.	Sheet rubber, double sided tape and work glove nitrile fingers are placed on the claws to enhance form closure. Under full work load, replace after 1–2 weeks.	Fits humanly worn normal work gloves such as nitrile work gloves to enhance form closure and grip. Under full work load, replace after 1–2 weeks.

^a^Not acceptable / not negotiable in work environment (e.g. due to item being costly or irreplaceable or hazardous or trace relevant or contaminant)

^b^Is performed just as well or better without prosthesis with result focus on task. VC: voluntary closing

^c^highlight best choice for terminal device (where applicable)

### Death scenes / field work

**Fig. 2:**
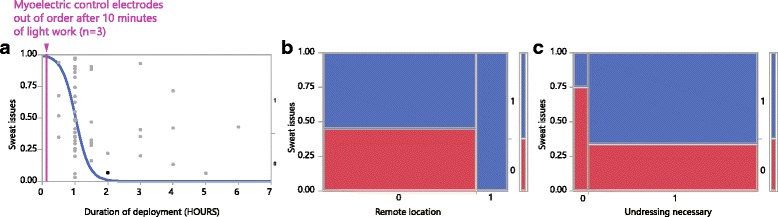
Occupational strains – **a**: Duration of deployment correlates with excessive sweat; myoelectric sensors start to usually fail due to sweat (purple line) after 10 min of bodily exertion, a third of a minimal duration of a death scene investigation; after 1 h working on scene, excessive sweating occurs in 50% of all cases and before 2 hours are reached in all cases; logistic regression (blue curve) indicates that excessive sweating occurs in over ∼ 85% of cases with duration of deployment over 1,5 hours; Chi-Square LR p < 0.001. **b**: Half of the non-remote but all of the remote locations generated excessive sweating (Chi Square LR p=0.0036). **c**: When undressing a body was a requirement, excessive sweating occurred in 60% of the cases but just in 25% when body was found naked (Fisher’s Exact Test: n.s

**Fig. 3:**
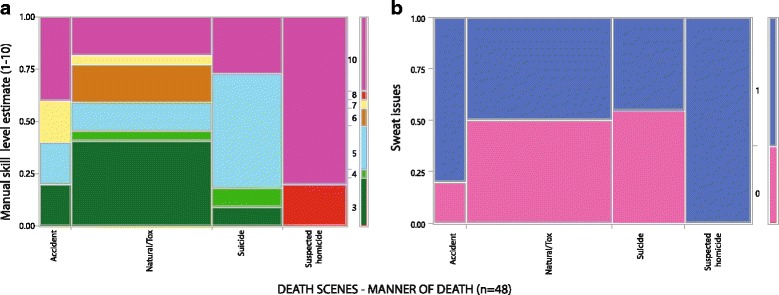
**a**: Manner of death (x-axis) correlates with required manual skill level (MSL) (y-axis; color code see right side of diagram) in that suspected homicide cases require a MSL of 8 to 10, whereas other manner of deaths range from 3 to 10; the differences between the manner of death categories with regard to MSL are statistically significant (Chi Square LR *p*=0.0013). **b**: Manner of death (x-axis) also significantly correlates with sweating being a significant workplace issue for suspected homicides (100%), accidents (80%) and others (about 50%). The differences between manner of death categories with regard to excessive sweating occurring are statistically significant (Chi Square LR *p*=0.005)

**Fig. 4:**
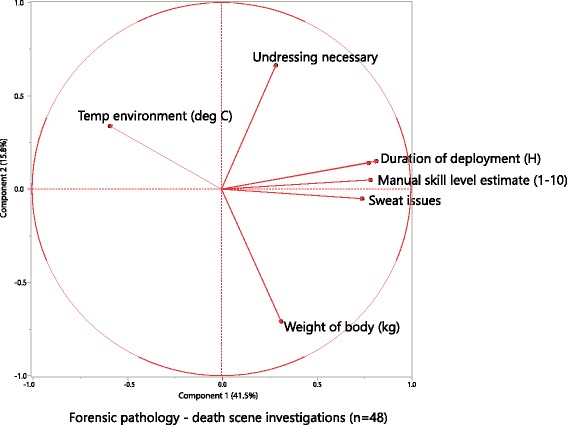
Principal Component Analysis (PCA) shows that the actual factors directly impacting excessive sweat causing soaked clothes are duration of deployment and manual skill level estimate. Ambient temperature correlates negatively with excessive sweating in that very cold death scenes are usually outside and do not always allow for an excessively differentiated clothing layer adaptation due to the nature of these scenes. Weight of body, and requirement to undress the body pale against these in comparison for the examiner in question

Retrospective evaluation of occupational aspects covered WS’ most recent 48 consecutive cases (notes, protocols). Duration of on-site work (deployment) ranged from 0.5 to 6 hours. Ambient temperature range was -14,5 to +30,3 deg C. Manner of death included mechanical violence with suspected homicide (10 cases), suicide (11), accident (5) and natural or poisoning (22). Weight of bodies was 77,5 (median; range: 50-130 kg). Manual handling of a body was aggravated by the body’s skin not being dry and clean (as it was in 21 cases): putrefaction (5 cases), charring/burning (1), significant amounts of blood (8) and slippery skin (13) due to various reasons (e.g., water). His own sweating being an actual issue for the investigator was noted where his clothes started to become soaked (dripping) (see Figs. 2, 3 and 4); there, breaks had to be taken to pour out excessive sweat from the prosthetic liner. Massive sweating for this protocol was defined as standard upper body clothing (two layers: T-shirt, long sleeve shirt) being still visibly wet after around 30 min after the effort. No easy to apply objective heat exposure rating exists, but subjective rating which we used for this report has been shown to be just as effective [50, 51]. Physical strain was typically not restricted only to WS in his role as field forensic pathologist: other specialists, such as forensic scientists, all working in protective gear, were usually soaked, too. A manual skill level (MSL) was subjectively judged to range from 1 to 10, with 10 being difficult given experience (similar to Task Difficulty in [52]). MSL has been rated higher for higher body weights, the presence of single pieces of evidence to be handled below ∼ 3 cm diameter, tight clothing on the body, narrow or tight space, and wet or slippery surfaces. Clean/dry bodies averaged an MSL of 4.2 versus an 8.3 score for non-clean/non-dry bodies (Wilcoxon *p*< 0.0001). Clean and dry bodies were substantially more frequently part of the natural or poisoning manner of death, whereas accidental, suicidal and suspected homicidal death cases (working categorization) contributed to significantly more bodies with a non-clean body surface (Chi-Square *p* = 0.009). The working categories of manners of death (in a sequence of decreasing average MSL) were suspected homicide, accident, suicide, and natural or poisoning (Fig. 3). Massive sweat issues occurred (in decreasing percentage of cases) in suspected homicides (100%), accidents (80%), suicides and natural or poisoning cases (about 50%). The necessity to undress a body was noted as a factor for all death scenes where the body was found not naked. All numerical results were non-normally distributed. Further documentation was obtained using a socket mounted video camera. That work caused collateral efforts, including exposure to relevant weekly laundry volumes. Additionally, clothing was state of the art professional work gear that was adapted to the environment, including shoes with safe soles and zipped with lock laces, battery heated switchable jackets for winter and evaluated protective clothing for critical death scenes [53].

### Office, research and laboratory work

(1) Typing: our department^6^ issues all forensic and medico-legal output, quality management, accreditation, correspondence, course and education related as well as scientific work, in typed-up form. This task may require 8 to 10 hours a day of constant text creation and editing. Voice recognition is not always an option. (2) Research and laboratory work also requires handling of small, slippery, expensive or fragile objects. These may comprise containers, substances, tissues, glass slides, cameras, scanners, microscopes, pump devices, large containers with special liquids, and at times must not be contaminated during manipulation. A “no failure” requirement exists for most grasp, transport or other manipulation tasks regarding falls or drops, spillage and other handling accidents such as contamination. (3) Meetings, presentations, negotiations. There, a prosthetic arm may be expected to “hide” the potentially visually offensive handicap, to reduce distraction and allow others to better focus on any relevant topics “at hand”.

### Mapping of forensic medicine tasks to academic tests of prosthetic arms

Outcome evaluation of prosthetic arms was performed with a focus to the PDW aspects outlined here. No previously published academic tests were employed, mainly because they do not adequately reflect the intense level of exposure in the user domain and are thus irrelevant for this application domain. The Carroll test specifies a range of objects (weight range 0,34 to 576 grams; size up to 10 cm) for manipulations as prosthetic gripper performance indicators [54], whereas for PDW, a wider weight range in a broader range of shapes are the norm. For the Carroll or SHAP test, the user is placed in a chair in front of a table whereas in PDW situations, manipulating or holding activities occur from various body positions and also dynamically. The descriptions of SHAP objects (coins, buttons, food, jar, jug, carton, tin, jar) [55] as well as clothespins [56] do not claim specified or standardized shapes, dimensions or forces. However, these tests also are largely irrelevant for many actual prosthetic arm use situations due to conceptual issues. That is not a common problem for all prosthetic limbs: with relevant device performances along just a few metric dimensions, prosthetic leg components can be evaluated with well-defined tests [57].

As opposed to that, ADL confront the daily prosthetic arm user with a complex range of non-formalized [58, 59] manifestations of cultural artifacts (e.g. [60, 61]; “endless variations” [62]; “(..) designs things that are easily and inexpensively manufactured” [63]). One particular example for a culturally and practically relevant ADL that evades technical standardization is the opening of a jar by turning its lid: the SHAP instructions state that “the lid should be placed on the empty glass jar and tightened only with sufficient force as would be expected for everyday use/self storage” [64]. Now, the televised footage of the CYBATHLON 2016 showed one pilot failing to open a jar lid using an iLimb prosthetic hand [8], so obviously, force is a key issue here. So, it will be of essence who closed the lid and how, with remaining uncertainty: lid closing or opening forces vary widely because when applied to jar lids, grip torque ranged between 0.86 and 6.56 Nm, across sex, age and grip type used [65, 66]. The high dimensionality of grasp-object-situation spaces thus may ideally be reduced for relevant (rather than highly collinear [67]) situation, grip and object specifications particularly in the context of work-specific prosthetic arm use [68, 69].

Relevance in the context of testing prosthetic arms meant for work integration can, logically, only apply to work relevant function. For the currently known clinical prosthetic arm tests, the test user is not explicitly designated or meant to sweat, to walk an hour carrying equipment beforehand, or to handle slippery bodies of a median weight of 77kg. Published tests also lack serious penalties of PDW. In real life, one cannot just drop expensive equipment such as cameras without expecting a relevant penalty. Thus, we applied a “not acceptable for this line of work” judgment for some performance failures (see Table 1). So the current lack of applicable scope with conceptual lack of relevant standardization imply that reports such as this – referring to what may seem to be non-standardized situations such as death scenes, office or lab work – will be at least equally relevant from a testing perspective for the interest groups related to prosthetic arms (see also “Discussion” section).

## Methods

### Description of problem domain

#### Body-powered technology

The medical care official of the employer was aware of the first author’s disability. Before user driven developments (see below) were initiated, we saw considerable problems in the work usage of prosthetic arms.

Work typical grip frequencies would wear down conventional cable sheaths with cable breaks, typically within 4 to 10 days, due to friction, also causing down time of a few days up to a week until repairs could be made. At that point, only terminal devices with manufacturer loaded springs (Otto Bock (OB: Otto Bock, Duderstadt, Germany) hands or split hooks) were used (and not devices with the option of user defined increase of grip strength and cable loading). Thus, the cables were only exposed to manufacturer limited loads.

The first two years after below elbow amputation in 2008 saw at least 46 prosthetist appointments of about 3 h each, including travel, mostly for cable repairs. On top, the user conducted a considerable number of cable replacements by himself. An informal survey across local and international prosthetists indicated that there was no current technical solution available to remedy cable shredding effectively, and no solution was available to order or even just to test.

Terminal devices and the wrist connector failed at various points. An OB double cable hand (model 8K24 [70]) irreversibly jammed within minutes of first use. An OB single cable hand (model 8K22) would break within a few months, and after replacement, with the same type of mechanism failure. An OB MovoWrist (model 10V39) irreversibly jammed within seconds of first use. An OB Rachetless Wrist Joint (model 10V10) with a spring fixing a threadless stud (model 10A44) would dilate over a few months and lose function. Various OB hook models exhibited a range of problems, containing temperature dependent jamming, hook joint wiggle, or breaks of steel cable connections. Terminal device adapter bolts did not exhibit the same diameters across instances but varied significantly, according to our own measurements (OB model 10A44), so some studs were not fastened, others jammed the wrist mentioned above (OB model 10V10).

The components had most likely not been designed for PDW. As one example, the wrist product sheet (OB model 10V10) [71] declared that the wrist component was intended for everyday use but not for extreme sports such as free-climbing. It had not become clear what the difference was between lifting part of another body’s weight (an everyday activity in forensic medicine) and part of one’s own body weight (climbing). An informal user survey showed that other commercial quick adapters for wrist units also tended to wiggle early into heavy use. A failing prosthetic wrist connector as weakest link, therefore, was a small part in a larger picture. Contact with commercial providers did not indicate availability of any better wrist adapters and thus initiated user driven development of a newly built wrist and adapter setup.

Wrist instability, carpal tunnel syndrome, double crush injury with plexus compression and episodes of lateral elbow epicondylitis of the anatomically intact arm warranted physiotherapy treatments. A dermatologist was involved in reviewing the liner and socket revisions required to address congestion eczema. Neurologist, orthopedic surgeon and radiologist consultations were performed as the user initiated the development of an insurance funded custom shoulder brace.

#### Myoelectric technology

Based on pre-evaluation, promotional and technical assertions, stump length, hand size and wrist connector considerations, a myoelectric iLimb Revolution model (Touch Bionics, Livingstone, UK) was acquired. Early tests using myoelectric technology had failed due to the hard socket not providing reliable skin contact when exceeding pull forces over ∼ 2kg, exacerbated by sweat. When the myoelectric socket was fit tightly for better slip control, excessive bruising occurred above the elbow. With very narrow liners or sockets, slips could be prevented, but severe stump pain ensued due to boney rather than soft tissue covered elbow region; also, socket-imposed elbow motion range restrictions rapidly caused shoulder muscle overuse. Extensive variations of technical suspension aspects with different hard socket designs or custom silicon liners were not successful during intensive development and testing over about four months. Dry skin [72] contributed to low myoelectric contact quality, requiring repeated re-adjustment with the liner skin interface during usage.

#### Funding

Insurance funding was obtained for both body-powered and myoelectric technology, including various custom silicone liners as well as custom-built shoulder brace and custom-built wrists. In addition, the first author so far privately invested about 6’000 CHF in myoelectric socket and technology trials and 2’000 CHF for supplementary body-powered components, supplementing insurance funding. Costs were kept down and time spent on revisions was low by re-using or scavenging of all those components whose life span exceeded the life span of the prosthetic arm as a whole [73]. User driven repairs evolved into user driven prototype designs. Also as a result of that, the initially integrated build and design of the body-powered prosthesis became modular.

#### Training aspects

There was considerable dedicated training particularly for the myoelectric arm, including a number of full day workshops for prosthetic use hosted by Balgrist Tec^7^. There was physiotherapist initiated home training, whereas whole areas - kitchen, laundry zones, garden work, car washing - were designated to train prosthetic use in a drill type fashion. Myoelectric arm training had been performed for about three years, body-powered use exceeded over four years in the current configuration.

Muscle power to provide the necessary grip, push or lift forces for body-powered technology was no issue. The first author keeps reasonably fit by performing regular and extensive sports routines. This effort is in line with performing a physically demanding job.

#### Choice of user driven innovations

Several prosthetic technicians, manufacturers, developers, and researchers had been contacted throughout the years in search for solutions for various problems described here. The first author of this study furthermore personally cooperated in a considerable number of prosthetic arm related research and development projects [74–81], also as a volunteer for feasibility or pre-test examinations.

Despite a wide ranging search, no solution to the failure of prosthetic components under PDW loads became apparent. User initiated development was chosen as the logical solution to address the known technological deficits [82], yielding the solutions specified here.

#### Prosthetic arm comparison, training and assessments

We compared a “bionic” myoelectric iLimb Revolution (Touch Bionics, Livingston, Great Britain) (TBI) and a customized body-powered arm (CBPA). The CBPA contains a number of new developments initiated or developed by the user, whereas the last author assembled the prostheses. Both systems underwent extensive trouble-shooting and problem resolution. On the job usage of both technologies over a few years was then supplemented with dedicated and focused intensive use of these devices for 12-14 hours a day for two weeks under PDW conditions.

During that period and later, due to various reasons, all work also had to be performed without prosthesis on, resulting in similar work exposure for non-use of a prosthetic arm (see Tables 1 and 2).

Grip strengths were measured with a Camry 200Lbs/90kg digital hand dynamometer (Camry, Kowloon, Hongkong). Noise assessment with mobile phone application in non shielded quiet cellar room on a soft padded sofa (base level <1 dB, mean difference to reference method ± 2 dBA [83]) (Sound Meter Pro 2.5.2, Smart Tools Co, on cell phone Huawei (Huawei Technologies, Shenzhen, Guangdong, China) under Android 7). Statistics and diagrams with JMP (SAS Institute, Cary, NC, USA). Socket side videos captured with Mobius ActionCam / Innoovv C3 camera (same camera type; Innovv, Hizhou City, Guangdong, China).

### Characterization off-shelf myoelectric technology / TBI problems

(TBI-1) sweat interference with the electrode function of the TBI with electrode malfunction after 10 minutes [84, 85] (3 trials, see Fig. 2 for context) and in context of sweat, decreased tendency of suspension to support heavy weight lifts or pulls; (TBI-2) glove durability: gloves would deteriorate to the point of requiring replacement as early as after 10 min of car washing [86, 87] or when left alone [88], without option to use gloves not issued by manufacturer [89]; (TBI-3) limb positioning interference with grip function causing inability to let go or hold grip [90, 91]; (TBI-4) general lack of reliable electrode function [85] and disconnection of electrodes with excessive pull [92]; (TBI-5) weak grip and weak hand (with low hand weight and low grip force being mutually exclusive constraints) [87] as issue for some (but not all) body transport or laboratory work; (TBI-6) lack of reliable precision grip and within-grip-activity change of grip configuration [87, 93] due to uncoordinated iLimb hand motors with the only synchronicity being simultaneous start and stop; (TBI-7) mechanical skin blisters after 10 hours of wearing the TBI for office work [72, 94]; (TBI-8) center of gravity (COG) too distally located causing painful shoulder and elbow tension after a few hours of typing already [87]; (TBI-9) irreconcilable use vs. warranty issues such as risk to inflict damaged cover, risk to use tools not “approved” by Touch Bionics, risk to exposure to moisture, dust or vibration [82, 89] and (TBI-10) problems with battery function at colder ambient temperatures ranging down to -15 deg C [95]; (TBI-11) Loud / irritating noise that distracts others (1) in meetings and (2) when working in the office or at home, emitting up to 72 dB [96, 97] and (TBI-12) use with a low degree of sweat but no control disruption over a whole day incurred electric burn type skin injuries with tiny blisters that took about six weeks to heal (encountered twice) [98–100].

### Characterization off-shelf body-powered technology/ CBPA problems

(CBPA-1) Cable tear down every 4-10 days using Otto Bock (OB) standard components [70, 87] with particularly rapid wear down of components of cable housing or sheaths. (CBPA-2) Prosthetic wrist unit spring dilation and insufficiently large diameter variation range of adapter componentry caused the start of an irritating wiggle after a few weeks and loss of bolt fixation after two to three months [87] (both cable and wrist are well-known points of failure of body-powered arms [82]). (CBPA-3) Nerve compression using figure-nine harness (F9H) after prolonged usage of grippers with high voluntary opening grip forces [101] causing carpal tunnel syndrome in a double-crush injury constellation (there, radiologic, orthopedic and neurological examinations were obtained) [102, 103] (Fig. 8). (CBPA-4) Friction/sweat rashes occurred every 1-2 weeks, forced a pause from wearing the prosthetic arm usually for 3-5 days and required treatment; Ossur Iceross Upper-X liners caused congestion eczema at the end of the stump due to a mismatch of a more cylindrical liner shape and a more conical stump shape [72]. (CBPA-5) Frequent deterioration of grippers of OB (hands, split hooks) caused repair down times. (CBPA-6) On top of the cable sheath as point-of-failure also orthopedic cable clamps (OB, 10Y3, the only cable clamp offered for both nylon and steel cables) appeared to prematurely damage steel control cables (21A 4=2), starting with early single wire breaks, to the point of sudden cable rupture usually within less than two weeks of use.

**Fig. 5:**
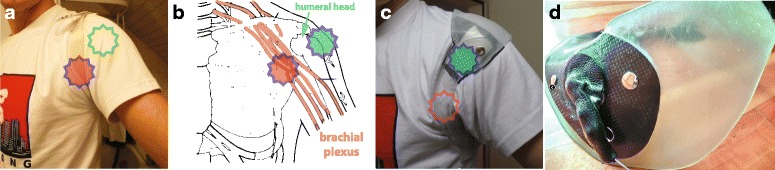
**a**: Conventional figure-nine harness (Otto Bock, Germany) compresses the brachial plexus (red star: compression point on brachial plexus, green star: humeral head and deltoid muscle for orientation; matching anatomy diagram in **b**) in what is a well known problem, also for backpacks. **b**: brachial plexus (nerve structures, highlighted red) with harness compression point (red star) in comparison with location of humeral head and compression point for shoulder anchor as shown in **c**. **c**, **d**: New development with a composite flexible thermoplastic EVA (ethyl-vinly acetate) and rigid carbon fiber shoulder anchor that effectively solves the problem by shifting the pressure point to the humeral head and deltoid muscle (green star) while relieving the brachial plexus (red star) by virtue of a rigid bridge

### Solutions for body-powered arm and user-driven modifications (CBPA)

**Fig. 6:**
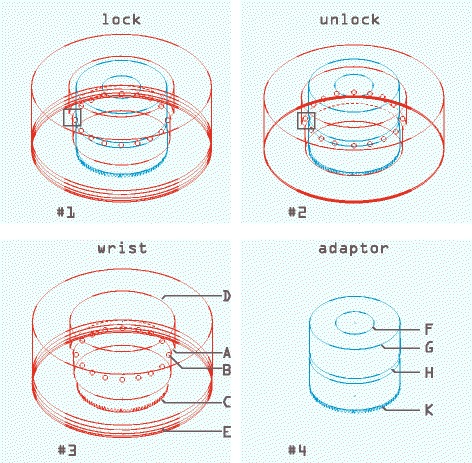
“PUPPCHEN” wrist – design details [106]: It contains one part, the wrist mount proper, that resides on the end of the prosthetic socket (#3) and a second part, an adaptor, that resides on the terminal device (#4). The design principle of the lock uses balls (#3, B). They hold the adaptor (#4) inside the socket-side wrist unit (#3) by residing in a circular groove of that adaptor (#4, H). The pressure on these balls force them inside that circular groove. That pressure can be released by turning the lock (#3, D) in such a way that a shoulder inside that lock (#3, A) is displaced so that the balls (#3, B) can slide back and release the adaptor (compare #1 and #2: black square). Rotation of the terminal device is prevented by interlocking the adaptor’s lower rim (#4, K) with a matching ring contained in the wrist (#3, C). The locking/unlocking switch (#3, D) is pushed up by virtue of springs at its base (#3, E). When unlocking the wrist (#2), these springs (#3, E) get squeezed

**Fig. 7:**
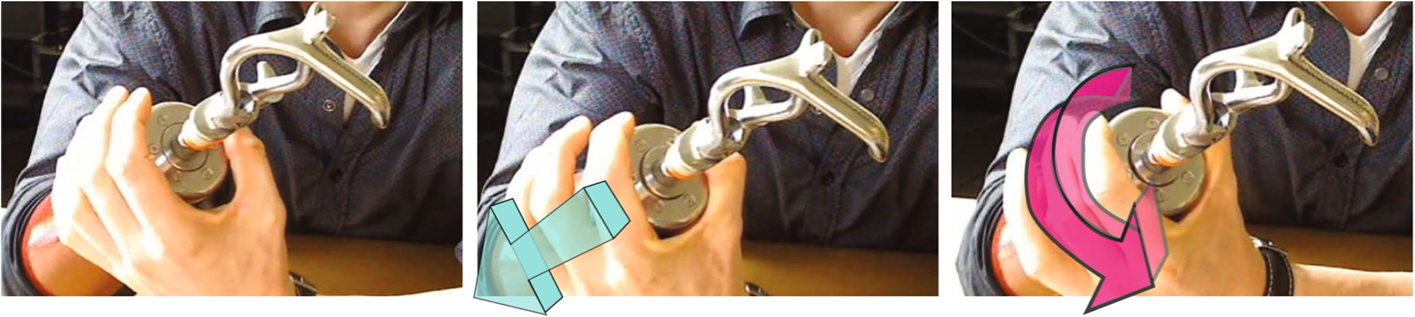
Opening the locked wrist lock. *Left image:* Grab wrist unit with a firm grip. *Middle image:* Pull wrist unit towards socket. *Right image:* Turn wrist unit to lock it in the ’open’ position

**Fig. 8:**
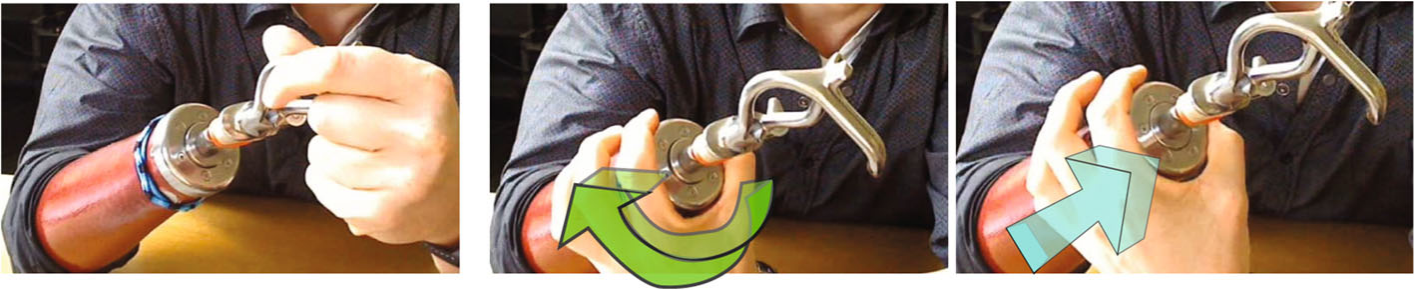
Changing terminal device position. Left image: pull out terminal device by a few millmeters. Turn it. Push it back in. – Closing the wrist lock. Middle image: Grab wrist unit. Turn it to allow it to slide back. Right image: Allow wrist unit to slide back. It is pushed into the ’locked’ position ny action of internal springs

**Fig. 9:**
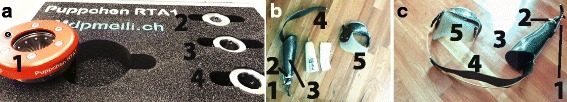
**a**: Wrist unit (diagram see Fig. 6, usage Figs. 7- 8) with socket mounted side (1) and terminal device adapters (2: UNF 1/2-20 threading; 3,4: Otto Bock sub-16mm diameter). **b**, **c**: assembled CBPA with (1) terminal device, (2) wrist, (3) carbon fiber socket, (4) cable, (5) shoulder mount/brace

**Fig. 10:**
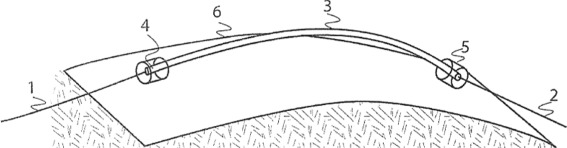
Bowden cable setup [105]: bendable but not stretchable element (6) on which two anchor points (4,5) are mounted between which the cable housing, sheath or conduit (3) for a cable (1,2) is placed so that any pull along the cable will forcedly press the endings of the sheath/conduit/housing (3) firmly into the anchor points (4,5) which as a design principle requires that the distance between the anchorpoints (4,5) is always smaller than the length of the conduit (3)

The CBPA was built with a carbon fiber socket, a pin-lock (Icelock, Ossur, Iceland), a lamination ring (OB 11D20=50), and a coupling used for quick connection of terminal devices (OB 21A7). Extensive user driven innovation was employed here (Figs. 5, 6, 7, 8, 9 and 10). A particular goal was increasing performance and reliability while reducing cost (which includes wear-down, repairs, replacements and down time). A modular rather than an integrated build was achieved to also allow for user accessibility, repairs, and component or part exchange. The cable control unit was fixed to the socket with just two screws. The cable or sheath exchange now can be done by the user without having to wear another prosthesis. (CBPA-1) The cable mount was modified to incorporate Bowden cable principle on flexible soft body curve shapes. The design introduced nonlinear rather than unilateral curved force distribution [104, 105], see Fig. 9 9 B/C and Fig. 10, with maximal cable force delivery exceeding 250N and unserviced operation of up to 9 months under described work conditions. Steel rather than perlon control cables were used for higher strength. Cable sheath material were Shimano pre-lubricated brake cable sheaths (Shimano, Osaka, Japan). At the end of the lifetime of the cable sheath, the cable deterioration would announce itself over hours or days rather than causing sudden rupture. For this, the sheath ends were regularly checked for visible sheath wire breaks. (CBPA-2) A quick lock steel wrist unit “PUPPCHEN” was developed after WS’ specifications [106] (Figs. 6, 7, 8 and 9) allowing an unserviced use of at least four years under described work conditions. A low profile fit was necessary to avoid excessive length. Wrist materials were aluminum (cover/lid) and Ramax (Uddeholm, Düsseldorf, Germany). Within the domain of withstanding hazardous conditions, a first prototype of our wrist featured a helicline mechanism; that was not sensitive to sand or dust, but only allowed for six discrete rotational settings. The current version is more sensitive to sand or dust, but allows for continuous rotational positions, while requiring cleaning of the lock mechanism after four years. (CBPA-3) A cast shape modeled shoulder anchor (CSMSA) was developed to avoid nerve compression typical for conventional F9H. The CSMSA shifted the harness pressure point from a soft compressible area of ∼ 15 *cm*^2^ in the anterior axillary fold overlying the brachial plexus to an area exceeding ∼ 60 *cm*^2^ overlying the less compressible deltoid muscle and shoulder. That decreased the required arm extension to open the gripper from around 12-15 cm (F9H) to around 5 cm [107] (Fig. 8). This significantly improved postural changes required to open a voluntary opening or close a voluntary closing device. It also allowed for relatively heavy overhead work. The carpal tunnel syndrome incurred by F9H afterward almost fully resolved by itself despite ongoing work. Insurance funding was obtained for this. – No particular innovation by the authors was necessary when improving upon the following problems: (CBPA-4) Suspension inherent complications (congestion or friction) on the stump were dramatically improved by modifying the liner layering. We employed a soft double layer whereas a tube gauze (Tubifast, Molnlycke, Norcross, Georgia, USA) is worn on the skin, underneath a gel liner (Ohio Willowwood Alpha liner), initiated by DE (white sock like parts in Fig. 9b). (CBPA-5) The improved device choice contained Hosmer split hooks (models 5, 5XA, 6(containing a user-tweak [108]); Hosmer, USA), V2P (Toughware PRX, USA), Adult Grip Prehensor 3s (TRS, Boulder, CO, USA) [40] and Becker hands (Becker Mechanical Hands, USA). Cooperation of WS with Bradley Veatch when developing the V2P [74]. Support by John Becker on grip force tuning issues of the Becker Hand [109, 110]. These devices were equipped with gripper surface modifications to enhance grip performance [111]. (CBPA-6) Consultation with wire mount specialists was obtained for adequate steel cable rigging. This included protection using softeners and avoiding sharp bends, corners, adequately sizing sling eyes and correctly mounting clips. In that context, orthopedic cable clamps (OB model 10Y3 [70]) were replaced with regular cable thimbles and cleats [112].

Revised cable sheath mounts and adequate steel cable rigging avoided shredding, extending un-serviced cable lifetimes to over nine months under full load. In conjunction with the shoulder anchor, grip strengths for both VC and VO devices were increased. All materials worn directly on the skin were chosen for optimized stain and odor behavior. A shift towards modular design shortened repair times from a week to 1-2 days for critical repairs.

**Fig. 11:**
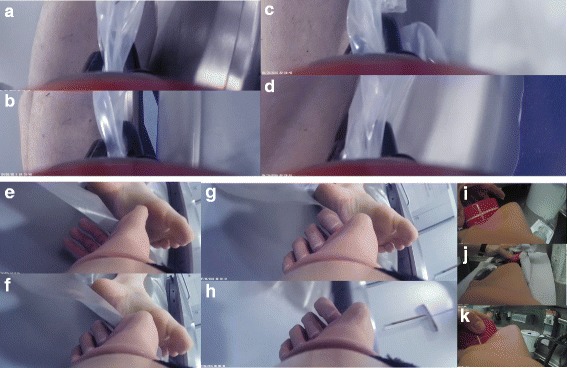
Socket mounted camera with video stills from workplace video documentation. CBPA (**a**-**d**) successfully grasps and holds on to plastic cover to pull body over from striker to CT table. TBI (**e**-**h**) can be seen to slip off not able to pull about 70 kg body weight by pulling plastic cover. Also, TBI fails to open jar with petrochemical substance (**i**-**h**) despite cleaning jar surface to make it less slippery

### Solution for myoelectric arm (TBI)

Ultimately, the TBI was mounted on an epoxy socket via a fixed wrist adapter due to length constraints (stump too long to fit a powered wrist) and a lanyard-fixed liner (Ohio Willowwood, USA) containing magnetic electrodes (Liberating Technologies, USA). Hard double layer sockets had failed due to thin skin around elbow and rapid extensive bruising. Custom liner solutions failed due to elasticity restrictions of processed materials (liners were either too narrow causing pain or too wide, not providing effective suspension). Battery placement was towards the elbow for a less distal COG. (TBI-7) Mechanical skin damage was mildly improved with the gel liner.

No further improvements resulted due to both inherent technology limitations (ILT) or manufacturer’s restrictions (MR): (TBI-1) Sweat interference and skin effects as well as ILT. Larger weight lifts or pulls causing shift or detachment of suspension ILT. (TBI-2) Glove durability ILT. There were no manufacturer approved durable gloves, whereas thicker gloves would significantly impede the already low grip power. (TBI-3) Postural interference ILT. (TBI-4) Lack of electrode reliability ILT. (TBI-5) Raw grip weakness ILT. A stronger hand would be even more excessive in weight. Preliminary tests with nitrile covered work glove were successful as to improving grip, but could not be sustained due to warranty restrictions MR (TBI-6) Grip issues due to both uncoordinated motor control and free thumb swivel MR. (TBI-8) Distal COG caused muscle strain problems ILT. This issue had been addressed by proximal battery placement already from the beginning. (TBI-9) Warranty issues MR. (TBI-10) Ambient temperature range was influencing battery function ILT. (TBI-11) Noise reduction was reported to resale agent but never remedied (thus classified MR). (TBI-12) No solution available ILT.

### Technology advantages weighed against each other

Posture and sweat interference for the TBI and grip geometry issues lead to failure of static holding requirements (TBI-3) in a workplace that generally is not set up to gracefully accept object drops.

The TBI suffered electrode malfunction and causes skin injury in sweaty situations which are a typical condition in this line of work (TBI-1, TBI-12). A weak grip force (TBI-9) was not always problematic, but it could be improved by using grip surface enhanced work gloves such as nitrile covered textiles. Due to warranty restrictions this was not a viable option.

Office work remained difficult due to mechanical damage of stump skin (TBI-7) when typing vigorously which, however, did not occur when wearing a tube gauze layered liner and light split hook of a body-powered arm (CBPA-4).

Physical and chemical exposure remained a tangible threat to the TBI hand that could only be equipped only with fragile gloves (TBI-2, TBI-9).

Pulling weight was not possible very well for some situations when handling bodies with the myoelectric arm (TBI-1, TBI-5) but could easily be achieved with an improved body-powered arm (CBPA-1, CBPA-2, CBPA-3, CBPA-4, CBPA-5).

TBI prosthetic arm malfunction often required a systematic troubleshooting approach [113] entailing access to and removal of the socket, fixing electrode positioning or re-placing liner. During PDW deployments and when wearing protective gear, that was disruptive and time consuming. CBPA advantages were a close, snug, swift and immediate feel to the dynamic integration of the prosthetic control into body motion and a reliable function under all work conditions, as predicted by physiotherapists.

An attentively observed and tested TBI property was not so much its adaptive grip (a body-powered mechanical Becker hand [109, 110] has that as well) but the option to define and switch grips. With the option to passively rotate the TBI thumb or set finger position, an extra practical advantage for the application of automatic grip switching did not emerge; however, the on/off-switch was used often to block hand configuration or grips.

### Other use than work and further developments

Once improved and tested for heavy, intense and extensive loads for workplace application, the resulting CBPA was also used for intensive applications outside work. It was used for bicycling (highlights include Stelvio pass, moutain bike trips also in deep winter with spike tyres, using various bicycle adapters), garden work (∼ 2 h over 35 deg C ambient temperatures) as well as transporting and mounting furniture (up to total weight of ∼ 550 kg materials with single package weight up to ∼ 55 kg). No damage to the CBPA or skin rashes were noted even for that type and extent of usage.

Both body-powered and myoelectric systems remain under further development regarding problem identification and solution, and both systems remain in regular use, although with different frequency. Both systems are undergoing further developments.

## Results

### Quantitative aspects

Grip strength of TBI ∼ 1,6kg. Grip strength of CBPA: Hosmer work hook ∼ 5kg, TRS Prehensor ∼ 25kg. Grip strength of anatomically intact hand ∼ 47kg (above upper tertile for bimanual males at similar age [114]).

TBI weight 1030g (895g socket, battery, wrist and terminal device; 135g liner with electrodes, COG ∼ 6 cm distal to stump end. CBPA weight 755g (630g socket, wrist and Hosmer model 5XA split hook device; 125g liner;), COG ∼ 3cm proximal of stump end. Weight of single CBPA terminal devices: TRS adult prehensor 3s: 393g; Hosmer 5XA: 139g.

### Qualitative aspects

Side by side comparison of overall usage experience (Table 1) and a more detailed terminal device appraisal (Table 2) shows that the CBPA provided more reliable, comfortable, powerful, light-weight, cost-effective service with less maintenance.

Most notably, CBPA grip reliability, grip force regulation, grip performance, center of balance, component wear-down, sweat and temperature independence as well as skin state were good. These results were mostly derived from wearing these devices for 12-14 hours a day for two weeks under PDW conditions while monitoring effects closely.

The option to switch VO and VC for body-powered arms introduced a breadth of control paradigms that was unmatched by myoelectric control. Terminal device swap from VO to VC control was preferably done after loading the car and driving to the location, and before going into examining a body (see also Table 2 for best terminal device performances). There was no overall single best terminal device for the CBPA if one optimizes for full performance. That was why the user driven wrist design had to allow for frequent quick swap of devices with full connector reliability. Increased grip performance by adding soft layers of materials weighed against the need of having to replace them frequently.

Overuse and asymmetry aspect: Severe shoulder and neck pain after office work (typing reports) with TBI already after 1 day; severe shoulder and neck pain using CBPA less accentuated, slowly building up over weeks and months under regular work loads, with about 4 critically painful days per year. Bi-manual support with body powered arm, particularly for heavy weight lift and handling, was supported best with VC device (see Table 2), resulting in perceptibly less tendon and muscle strain for the anatomically intact arm. Some laboratory work when assembling a series of device parts was further supported with a bench vice. Left arm wrist: chronic instability, pain severe after heavy one-armed lifting, significantly better when being able to use prosthetic arm. Left arm elbow: lateral epicondylitis, best addressed by sharing load for many repetitive tasks across both arms.

Skin: Blisters and rashes with relatively long healing time (up to 6 weeks) for TBI; rapidly healing friction rash with CBPA slowly building up over continuous heavy use after 4-5 work days with definitive need for a break of 2-3 days after continuous use of about 10-14 days.

Temperature: no control failure for CBPA even at very cold ambient temperatures.

## Discussion

We initially encountered severe problems with both current body-powered and myoelectric technology. These were found to be not unusual as a general consumer experience. Subsequent prosthetic arm rejection is a logical and typical user reaction [115, 116]. Myoelectric technology may have a relevant role in supporting amputees with restricted capabilities to drive body-powered arms, including higher level amputations. But as stated initially, this article addresses the requirements for a particular intense work application from view of a UBEA.

In this instance, expert user driven innovations under permanent, intense and continuous testing on the job [117] resulted in the necessary improvements to achieve such a prosthesis.

### Consideration of requirements of different interest groups

Tense contrasts exist between promises, hopes or predicted failures on the one hand, and technical realities on the other hand. The fact that current myoelectric technology lost the Arm Prosthesis Race of the CYBATHLON 2016 against body-powered technology contrasts with high hopes and promises going with the new “bionic” hands [7, 42]. The fact that body-powered prosthetic split hooks can be powerful prosthetic aids contrasts with the problem that they are vilified [32, 118, 119]. These contrasts affect various interest groups that relate to prosthetic arms differently.

UBEA often find that for ADL or light work, the stump is the best prosthesis [120]. In the age group 2–20 years, UBEA without prosthetic arm outperformed both wearers of prosthetic arms as well as people without disability for ADL across freely distributed bimanual tasks [121, 122]. Prosthetic arms are not of proven value to help psychosocial adjustments [123]. Moreover, arm amputees may regard not wearing a prosthetic arm as part of affirming a public image of different ability [124] particularly in the light of social pressure. The prevalent non-usage of prosthetic arms may be the best functional, economical, proudest and thus rational choice for ADL and light to moderate work [10].

Users that expose themselves to their devices may end up as the ultimate experts [4, 118]. They try to get their consumers’ complaints to be taken seriously, but there may be powerful social and neurological mechanisms that prevent this [125]. The bare arm amputee risks to upset others visually so much [119, 126], that expensive gadgets have now shifted towards the center of a sociological demarcation process [127]. Thereby, societal mechanisms exert a strong push towards amputees to stereotypically cover their stigma [126, 128]. Conversely, the few amputees that do feel personally concerned by that push may offer to comply with that request by exclusively accepting expensive or futuristic-looking rather than functional technology [129–131]. Within that discourse, raw mechanical functionality risks to deteriorate from being a core property to being, at best, a superficial label, while affinity-driven product ratings [132] may risk to distort public perception of their advertised (but not actual) technical performance. In a further twist of society attributing stereotypes, amputees wearing “bionic” hands risk to be perceived as “cold” and as “high-tech”, and thus as a social threat [133].

Families of amputees or prosthetic technicians have assumptions regarding the role of prosthetic arms that differ from those of amputees [123], as do engineers [134]. Current prosthetic arm research and development mainly focuses on myoelectric [118, 135, 136] technology and, more recently, 3D-printing [137]. If nothing else, these devices are marketed to conform to the requirement of a social standard of costly modern technology [32]. Myoelectric and 3D-printed arms are thus assumed to support at least light work or ADL. But only 23% of the users rated the weight of a myoelectric hand as acceptable [138]. Only 12% of the male users found the noise of their myoelectric hand to be not disturbing [138]. Usage of myoelectric arm was indicated most often for using cutlery (76% of men), handicrafts and even opening/closing doors (71%) [138].

A more definite role for myoelectric arms to play particularly in UBEAs’ lives may thus depend on what real needs this new technology manages to cover [128, 136, 139]. However, the list of known issues relating to current myoelectric arms, remains long. It contains electrode related skin rashes [98, 99], sweat interference with electrode functioning [84], postural interference [140], high weight and distal center of gravity, insufficient durability [47], noisy distraction [141], absent proprioceptive feedback [142], uncoordinated grips [93], fragile prosthetic gloves [143], extreme costs [144] and unattractive appearance [45, 145].

Arm amputees with PDW to deliver are far more constrained regarding the choice of their prosthetic arm build, controls or components: they will more likely have a vital need for prosthetic arms that function, also under harsh conditions. Body-powered arms also dominate the market of prosthetic arms that are indispensable for PDW as well as sports [3, 44, 146]. For work with occupational heat exposure, biological or chemical hazards, large weights or widely ranging ambient temperatures, there is no other technology. Development of body-powered technology currently is only conducted by a small number of individuals and groups (e.g., Randall Alley [147, 148], Bob Radocy [40, 149], Bradley Veatch [74, 150, 151], Dick Plettenburg’s group [152–156], Aaron Dollar’s group [157] and John Sensinger’s group [158]).

According to our results, even some of the current commercially available body-powered components are nowhere near sufficient for PDW as outlined here. When facing such a situation as a consumer, discarding the faulty product is a far more likely reaction than trying to fix it, which can be very difficult [159]. We worked on two fronts for that: we tried to optimize both body-powered and myoelectric technology, both within the available options.

Approaching solutions for a PDW workplace from a general development and research position, one will consider that most of the hazardous, intense, sweaty or manually challenging work aspects cannot be changed [160, 161]. Also in the future, decomposing, heavy and slippery bodies will be found, also in narrow confined spaces, also of messy premises. Also for years to come, lifting, retrieving, turning, undressing and examining bodies in such situations will remain strenuous and require tough, light weight, durable prosthetic arm components with high tensile and compressive strengths for the experts that perform these duties. Occupational tasks of this specialized and individual nature will require concessions and compromises also concerning posture [160]. One may have to work out regularly to achieve and maintain fitness for such work [162]. Long and drawn out death scene examinations when wearing protective gear have aspects of “mini-expedition” style missions: one goes in, then one is in there under full strain, with executive and manual challenges and responsibilities, without any easy option to exit or troubleshoot, until only hours later, when that mission is over. And so there are other instances where equipment has to conform to harsh occupational requirements, and equipment specifications seem not too different: for large expeditions, reducing weight, improving performance and extending longevity of equipment can attain game changing significance [163]. So, research and development has proven, elsewhere, that it can understand and integrate such concepts outside the circle of amputee problems.

### Narrowing technical options

An ideal mission-critical design [164] – as a necessary property for a prosthetic arm – will deliver reliable and largely error-free performance that at least approximates industrial quality standards as well as delivering performance across the specified exposure. A conformant prosthetic arm is built to minimize ill side effects, bodily injury or damage. It is built with a modular design that allows fast user repairs with widely available and affordable materials. It offers protection from overuse in the light of bodily asymmetry and heavy bi-manual work [17, 19–22].

Studies that discuss prosthetic use and overuse never normalize or stratify for actual work exposure, prosthetic arm proficiency for intense work, and actually delivered manual work. In our case, a supportive prosthetic arm allowed to perform hard work at the same functional level as peers, whereas a wrong design would cause severe shoulder pains after 1 day of regular typing work.

Mission-critical design requirements are not met by some of the current prosthetic parts that we encountered. Clinically relevant side-effects are a reason to reconsider design aspects of a prosthetic arm once lesions take too long to heal or when they risk causing permanent damage. Sudden or erratic failure while wearing a prosthetic arm can be a dramatic and stressful event; this is remedied by pushing a system to exhibit graceful degradation, which gives the user time to intervene.

Body-powered prosthetic arms are very intuitive to use. But actual motor skills including fine motor skills are acquired only by sufficiently specific and sufficiently extensive training [165, 166]. To no surprise, absent proficiency of large shoulder and trunk muscles to perform fine grasps with a body-powered control in untrained non-amputees causes their control attempts to deteriorate at higher pinch forces in a study that makes a great case for training [156]. Also, absent sufficient specific training appeared to be the reason of fatigue in most non-using amputees when trying out body-powered arms, whereas the only actual daily user of a body-powered arm in that case series did not exhibit any significant restriction (study subject number seven [167]). The first user of this study had therefore been advised by his physiotherapists early on, to not just try out body-powered technology, but to really wear it for a few years. Ultimately, large arm, shoulder and trunk musculature may be trained for heavy lifting and subsequent fine control even more efficiently than hand muscles [168]. Conversely, electric motors or batteries may simply be dead weight for a UBEA that delivers PDW over years and that has sufficiently extensive and sufficiently specific strength to provide forceful body-powered grips.

#### Dermatological side-effects of prosthetic arms

Friction rashes are a frequent side-effect of wearing a prosthesis [72]. Conventionally, polyurethane or silicone liners are worn directly on the skin. When sweat disrupts close liner adherence to the skin, the sweat soaked outer layers of the skin will easily abrade and develop a rash or blisters, as early as after a few hours. It may take days for a rash or blisters to heal, during which the prosthesis should not be worn. Tight cotton is known to effectively treat ’acne mechanica’ in soccer players [169]. We employed tight tubular gauze to be worn under a gel liner. It interfaces with the skin through micro-compression by way of many tiny fabric strands. These swell up to a degree as sweat fills up the cotton, while the outer skin layers remain relatively dry [170]. With a body-powered arm, the socket does not contain electrodes that sit on the skin and provide ridges where soaked soft skin layers risk to get abraded. So protection from friction rashes can allow for far greater exposure under sweating with a body-powered arm.

Skin burns are not uncommon to develop in the vicinity of myoelectrodes [98, 99]. Here and under our observation, these lesions came about under moderate amounts of sweat that had not acutely disrupted myoelectric control and took about four to six weeks to heal. As described elsewhere, we also observed blister configurations as part of these burns. The underlying technical aspects of these burns appear to also affect implanted electrodes [171]. Furthermore, heavy sweating would disrupt myoelectric control as early as 10 min into PDW [84]. Research into non-electric modes of control of devices as so far yielded both subcutaneous [78] as well as surface shape [172, 173] derived control signals as viable alternatives, at least from an academic research angle. From a PDW aspect, too much equipment is not a practical option [174]. With regard to skin preservation under PDW conditions, we found that body-powered suspensions could be coerced to conform best.

Typing contains its own perils. A long duration of repetitive small stroke actions can be hazardous, so even small differences in weight amount to large effects at the end of a day. Myoelectrodes’ ridge structures pressing into the skin caused a significant friction rash and large blisters, just after one day in the office with typing work. The socket will experience larger repetitive motions also due to a higher myoelectric terminal device weight. A tightly fitted body-powered configuration with a light aluminum split hook performs with less amplitude and less momentum. This is the case particularly with deadline work and long hours of writing [175].

#### Sudden failure rather than graceful degradation

Graceful degradation of performance even under adverse conditions is essential for mission-critical reliability [164]. Research and development will have to address this aspect consciously.

A predictable grip geometry is required for efficient forward-planning of dynamic push-release or reach-grasp trajectories. A multi-articulated hand that lacks finger tip coordination cannot guarantee a reliably repeatable grip configuration [176]. Lack of geometry control invariably will cause grip failure that may surprise the user, causing “sudden” or at least unexpected problems on a functional level, as seen at the CYBATHLON 2016, where a rigid gripper with just two claws outperformed some of the demonstrated multi-articulated hands due to this problem [7, 177]. Plannable grips so far benefit from rigid or constrained grip geometries. This to a degree may explain the various split hooks’ models success within amputees [41–44, 146]. The design of multi-articulated hands could possibly be improved, as researchers have identified and understood this problem [93].

Posture or stump position may negatively interfere with myoelectric control. Even professional training levels and trained controlled circumstances cannot prevent sudden occurrences of this phenomenon [7]. Typical myoelectric control uses two electrodes to control a single degree of freedom. They are placed on the flexor and extensor locations with best signal-to-noise ratio. Incidentally, these locations typically contain muscles that are also activated during elbow flexion, extension, or during stump pronation or supination, regardless whether the user intends to open or close the myoelectric device. Flexing the elbow, standing up or changing the position of the torso while keeping the hand in a constant position (which will entail elbow extension or flexion) or other changes in the limb position risk to trigger unintended signals [91]. Different stump positions are also known to interfere with multi electrode control [140]. This problem results from employing intrinsically polyvalent muscle groups for single function controls [90]. Especially when the user is distracted, and during dynamic work, this can drive up myoelectric performance error rates fast. While body-powered arms exploit posture of elbow, shoulders and back to directly transmit their shape change to achieve an analog cable tension actuation, myoelectric arms exploit polyvalent forearm muscles for digital single function control in UBEA.

It is thus fair to say that myoelectric arms are or can be also, to a degree, body-powered [178]. The art consists in making that a wilful and consciously controlled act. With that, there are two distinct differences to proper body-powered control. In body-powered arms, cable tension is built up gradually, and there is considerable proprioception of the analog control state, to a degree where body-powered VC devices can be used to precisely vary grip power from very subtle [27] all the way to over 200N. Myoelectric arms lack an analog proprioception across any control range. Secondly, the muscles used for body-powered control allow for a relatively intuitive separation of gripper actuation versus limb position change. As a key property of the control system, it results that body-powered control degrades far more gracefully when changing limb or body position. The user always feels the cable tension. While it is a training paradigm that myoelectric arms allow for precise and fluid motions [179], we found that controlled stop-and-go procedures can be more effective to prevent the limb position effect.

With both myoelectric and body-powered systems following bodily motions, both can be thus used in a freestyle way, or ’tricked’. One useful posture trick, given conventional myoelectric systems, is for the user to not at all move the stump, elbow or shoulder while performing critical grip maneuvers. An elevated shoulder and stiff elbow in an attempt to avoid posture effects will eventually cause overuse symptoms on the shoulder and neck of the amputated side, but may be relatively efficient when carrying valuable items [178]. Another useful trick, for both body-powered and myoelectric controls, is to switch off or let go of the prosthetic actuation entirely, to avoid any postural interference with the gripper.

This has been the solution for the winner during the hot wire loop test at the CYBATHLON 2016 [7]: the pilot locked down his body-powered VC system’s control cable [180] before he started with the hot wire test. He was then free to focus on the loop position fully. He only unlocked the cable afterward. The other competitors did not appear to have visibly incorporated that body-powered aspect into their myoelectric race strategy [8].

Immediacy and option to manually intervene in real time, at every step of a manipulation, is far easier with body-powered arms. Being in full control over one’s own work pace is a key factor in successfully delivering PDW [181]. Manual overrides or visual signals could be added to myoelectric devices with little extra weight. Overall, due to a very intimate link between cable tension, proprioception and terminal device actuation, we found that a body-powered control was always far more reliable than a myoelectric system.

#### Grip quality and grip strength

Soft covers of grip devices are a relevant issue [111]: in the presence of friction, form closure of any object places less emphasis on the grip geometry (gripper shape, number of fingers or claws). There exists a negative relationship between softness and longevity of a gripper surface [182]. The softer the surface, the firmer an object may be held even at low grip forces, but the more frequently it decays and needs to be replaced. Then, user accessibility and very affordable materials become a critical issue.

For prosthetic hands, soft covers are typically gloves. The durability of gloves is important; it was mentioned as a relevant factor already in 1980 [143]. The constraints that exist are manifold: Firstly, manufacturers of prosthetic hands make narrow specifications for allowed gloves. Secondly, gloves mechanically impede actuation [183], so weak prosthetic hands are equipped with thin and fragile gloves. Thirdly, perforating damage usually calls for an immediate stop to usage as gloves protect the hand from dirt or fluid. With myoelectric hands being rather weak and heavy already, hand geometries deviate from a normal human hand in efforts to maximize efficient grip geometry. That again makes it hard or impossible to fit these hands with normal gloves that fit normal human anatomical hands. The softer the glove, the better the grip but the faster it is damaged [182] and needs replacement. Humanly proportioned gloves are mass produced at a wide range of makes and qualities for relatively low prices. Any terminal device that works without these constraints is at a clear advantage.

There is one adaptively gripping very precise and robust prosthetic hand that excels there. The body-powered Becker hand [109, 110, 184] is a very affordable, robust body-powered hand with a reliable precision grip and an adaptive grip, that fits standard gloves including regular work gloves, including those sold at convenience or hardware stores. Its mechanical design is technically very evolved. It is not clear why the 3d-printing community, that claims to search for affordable durable solutions with respect to prosthetic hands, has not identified that hand as an answer to their quest.

Equipping a standard metal split hook with silicone tubing or cutting sheet rubber to fit a V2P or TRS prehensor device is fast, supported by warranty regulations, and easy to perform for the user.

### Considerations about testing and reliability

Proper testing procedures will automatically pave the correct way for component development. Our initial negative experience with some of the currently available conventional prosthetic components may be seen as a clear reflection of current testing and product development practice. While we did provide our own relevant user driven device improvements, we would never have identified the need for them, and we would never have refined them to their current performance level, without PDW application. This forced us to address obvious conflicts between reality and expectation.

#### Popularized testing

From a hard working user’s perspective, a prosthetic arm always has to serve a user’s occupational needs first. That is also the typical insurance perspective. Competitive challenges that serve these specific requirements will have to be accompanied by occupational therapy and professional task coach instructions, allow for sufficient training, allow for several repetitions with different approaches, also without the prosthesis on, and allow for a range of quantitative and qualitative job- and outcome relevant metrics.

An awkwardly positioned body posture for a few tasks scattered across a daily time line is of absolutely no concern whereas repetitive or heavy tasks require more focus on correct posture – a distinction currently absent from the literature [178]. Performance evaluations with an academic entitlement may require a fuller effort to document and evaluate control, grip, posture, failure and other performance characteristics across all pilots’ attempts. One will expect registration markers and multi-angle cameras [178] on every contestant, and several runs with the same contestants but different prostheses. There will be control runs with the contestants without prostheses and non-disabled controls. Sensible rating may be conceptually difficult as time is often of no actual concern, nor will an arbitrary pre-defined task or arbitrary weight leveling for bi-manual tasks be of relevance to many PDW situations.

Popularized entertainment style prosthetic comparisons [7, 55] could be re-defined, to cover at least some of these aspects. Even despite the CYBATHLON 2016 focus on comparing arm amputees’ performances related to activities daily living (ADL) “as entertainment” [185], more intense work could be additionally popularized, for example as an added CYBATHLON 2016 “lumberjack” show [186].

#### Occupational task oriented testing – lowering error rates towards “Six Sigma”

The usual ADL focus of occupational therapy [187] has not been shown to effectively facilitate PDW rehabilitation [25, 188]. Prosthetic arm testing so far avoids heavy or highly repetitive bi-manual work specific tasks including performance under sweat [189]. Upper extremity prosthesis user satisfaction surveys, while sometimes employing academic test tools such as the DASH inventory, SHAP or Box and Block test, systematically omit relevant details regarding their research subjects’ profession, job or occupation [190–193]. Hazardous conditions and large slippery objects are lacking; there is not even a true-to-life secretary typing contest for arm amputees.

Relevant testing in any laboratory setting will have to approximate PDW style tasks, just as testing people or equipment for space missions entail well engineered simulations [162, 194]. From a PDW user view, the functional focus may be on safe, secure, fluid and uninterrupted completion of difficult bi-manual work tasks. Lifting tests, for example, may focus on weighty slippery objects such as lifting oily sheet metal, lifting tasks encountered in forensic medicine, or lifting a large heavy box. Holding and handling tests may focus on chunky but valuable or fragile equipment, such as large mirror reflex cameras or laptops including cabling, as well as small and delicate items [1, 195]. Realistic exposure parameters for a wider range of work can be found in the literature; a larger survey showed that an average (but not maximal) weight for carrying, lifting, lowering and pushing objects ranges around 20–25 kg [11] across industries.

The current practice has not generated particularly reliable prosthetic arms: the published error rates are high. Researchers currently view conventional laboratory derived myoelectric control success rates in excess of 90% [196] or 96% [197] as good. Industrial manufacturing that is oriented toward workmanship and production [198, 199] defines acceptable failure rates around the “six sigma” to “nine sigma” range. And simple calculations will show just how relevant these figures are even for ADL in a home setting: unloading as few as 12 cups a day from a dish washer at home will amount to ∼ 360 grips per month. A grip success rate of only ∼ 99,7% will see one crashed cup a month, or a total of 12 crashed cups a year. Not even that may be sufficient for realistic industrial or even ADL application from the viewpoint of amputees, coworkers, employers or families. For industrial exposure, as in washing dishes for a restaurant, handling 1200 pieces of dishes per day may be a low figure; there, dropping one dish per month requires a grip success rate of 99,997%. Implementing industry grade failure rates for prosthetic arm component development and testing will be a first step into the right direction [200]. Once prosthetic arm systems exceed a “six sigma” standard under all work conditions (failed grips not in excess of 3.4/1,000,000, success rate exceeding 99,9996%), amputees may feel more interested in wearing one. Sensible advertising to critical customers may benefit from added quality ratings [201], particularly if they base on intense, strict and independent testing.

Private interests of arm amputees may cause their prosthetic arms to also require significant reliability and stability. In one arm amputee related private internet support forum^8^, the last consecutive 29 posts mentioned strenuous physical activities and related prosthetic issues (8 proud posts), motivation and discrimination aspects (8 posts), general queries (8 posts) and welcome notices for new members (5). There was no single reference to “bionic” prostheses. This points to the fact that privately initiated strenuous sweaty and hard activities are relevant within that community. For climbing, bike riding and other sports with a clear need for bi-manual work, frequent sudden failure is not an acceptable mode of product decay [202]. It goes with the territory that a modular prosthetic arm that conforms to sensibly low industrial failure rates also will be good for sports.

Even to just succeed in an expectedly low-intensity line of work or ADL of everyday life, a prosthetic arm that is built for PDW may be the one to use. In everyday reality, gradual escalation of any laboratory conformant and controlled environment type ADL situation may easily lead to any type of intense situation with a then failing prosthesis, whether staged or real [7, 203]. Due to escalating circumstances deviating from a dry stump skin and controlled sedentary position, myoelectric prostheses thus tend to perform worse than body-powered arms even during what one may call “normal life”.

### Building effective solutions

#### Shoulder brace

A regular figure-nine harness compressed the brachial plexus significantly and thus was found to be ill-designed for heavy long term use [101]. We thus devised a shoulder anchor. With both flexible non-distensible as well as rigid materials, the pressure is distributed across a less compressible and larger shoulder area, away from the brachial plexus. In combination with reduced compression of body tissues, this design reduced control cable excursion from previously 12–15 cm to around 5 cm. With that, the distance from the cable being fully relaxed to the terminal device being fully actuated was reduced to less than half. The choice of shape and material also stopped the brace from rotating its pivot point to the direction of the cable pull. That qualitatively increased the range of comfortably achievable postures, also including overhead work. Features characterizing our improvements of our customized shoulder anchor over a figure-nine harness were identified and confirmed robotically [204]. A similar design had been developed previously, with high acceptance by the users [205]. Significant posture improvements, particularly for demanding and repetitive work, are of known high relevance [206].

#### Cable sheath – sudden failure versus graceful degradation

Sudden cable failure as any other sudden device failure dramatically generates and perpetuates user dissatisfaction [116, 207]. Better planning for cable failure, therefore, became a priority. Both far more robust design and graceful degradation were made part of a mission-critical property of the prosthetic arm.

Replacing orthopedic cable clamps with correct rigging [112] entirely removed one source of frequent cable breaks. Conventional prosthetic cable mounts were found to suffer unilateral housing damage very fast and early, which then lead steel cables to break. User driven cable housing revision with a Bowden sheath fixation on a flexible belt extended the service-free life time span of the steel cable, from 4 to 10 days to over nine months, under higher actuation forces.

Also, the cable sheath revision opened up a far greater grip strength range: with reduced overall sheath resistance, more subtle control became possible. Cable shredding in prosthetic arms had previously not been solved [150, 208], despite space exploration relevance [209]. Our current cable mounts are made from relatively soft plastic, allowing for graceful degradation and a visual check of cable sheath status. Further mount designs improvements may see a replacement of conventional bicycle housing with stacked cylindrical shells [210]. Further functional improvements may entail loop routing [211].

#### Quick lock wrist

We experienced several commercial wrist products failing over work related tasks as outlined here. The problem of a dilating spring fixing a connector bolt was that of an overly graceful degradation: the amount of wiggle this wrist exhibited after a few weeks was irritating, but not sufficient to warrant full replacement. Wearing a device that is in its late stages of failure but not broken enough to pay for replacement, here due to excessive wiggles, may also be a rather irritating problem.

Technical wrist connector design also defines its failure characteristic. Our design extends the operative range towards pulling work-specific relevant weights without risking wrist connector wiggle, dilation or damage [11] while it is also constructed to withstand considerably higher weights. With that, it allows for heavy lifting as well as quick rotational angle or terminal device change.

### Further research and development

#### Cosmetic prosthetic arms

Within the realm of appearance appraisal, hands have a peculiar place [212]. So socially, the common treatment of an arm amputee wearing an obvious prosthesis does not seem different from the one that does not wear one [126]. Only successfully hiding the handicap stands a chance to effectively upgrade the amputee’s outcast status, if only from “discredited” to “discreditable” [213]. Currently, arm amputees are always exposed. A prosthesis that effectively hides the handicap both statically and dynamically does not exist currently.

Technically, the ultimate challenge for a prosthetic arm design based on a clear user need remains covering up the handicap effectively. Neither industry or research have achieved technology necessary for successfully hiding an arm amputation with a prosthesis. This may be an important next step in an attempt of prosthetic manufacturers’ to bring down staggering rejection rates. From the user perspective at the moment, the fact that no prosthesis conceals the disability usually ends up obviating a need for wearing a conventional prosthetic arm particularly if its gains are, weighted for hassle, effort and discomfort, marginal at best. Unforgiving appearance testing is required to facilitate research and development to steer towards actual “cosmetic” prostheses [214].

#### Functional prosthetic arms

Functional prostheses have their established role in hazardous bi-manual work, PDW or blue collar occupations as well as sports. As UBEA (without prosthetic arm) even outperform non-disabled competitors in typical ADL type bi-manual tasks [121, 122], testing and research may have to learn more about bi-manual task completion for that group, and if only to get a useful baseline.

Body-powered technology is sufficiently evolved that it can be seen as the key to unlocking the market for functional prosthetic arms. It can be built to offer reliable performance with graceful grip degradation, full integration of controls with body posture and minimal medical side-effects at relatively low cost. Current problems with fragile commercial components are easy to overcome conceptually, and we showed that practical solutions work under real conditions. To achieve this on a larger scale, mission-critical performance rates will have to be targeted. Targeted reliability for professional prostheses should lie in the range of fewer than 3 errors for a million single grips under all usage conditions.

Only with hard real world testing under sweaty conditions for weeks or months (to monitor skin and overuse) per test series will prosthetic manufacturers and researchers learn which control and gripper systems work well. Mild and cautious ADL are not suitable as target for testing, development and trouble-shooting functional prosthetic arms.

For any grippers, very affordable, easy to mount grip surface covers that are soft and resilient are the current challenge.

We also found that optimal usage entailed a relatively frequent switch of terminal devices, most notably between the VC and VO control type. For PDW under such conditions, the next frontier is thus in perfecting the design of body-powered heavy duty devices that contain a switchable VO/VC control [151, 158].

## Conclusions

Trying to build a prosthetic arm that works for work in forensic medicine taught us a number of things about current technology and its potential.

Many current prosthetic arm tests and rehabilitation efforts focus on ADL. That focus is not sufficient to achieve satisfying solutions, particularly for PDW.

For the purpose of developing prosthetic arms for heavy and intense applications, really demanding testing procedures are mandatory. Occupational challenges may then necessitate prosthetic adjustments that only appear once prosthetic devices are subjected to sufficiently hard tests. Control error rates of prosthetic devices need to achieve realistically low figures in the six to nine sigma range, as is standard across industry elsewhere.

Once heavy and intense work, robust control under sweat generating conditions and very low error rates are set as requirements, it will become a lot easier to identify viable technologies.

We found that initially, no currently available technology fulfilled these requirements. But body-powered controls could be brought to useful function with extensive user driven innovation and design, whereas myoelectric technology could not.

Once a prosthetic arm manages to cover demanding occupational tasks with low failure rates, ADL may not be an issue at all any longer. This was exemplified in the CYBATHLON 2016’s Arm Prosthesis Race, where the pilot with a “light” version of a body-powered prehensor won on a set of ADL-derived tasks against all myoelectric competitors.

## Endnotes

## Abbreviations

ADL: Activities of daily living. These usually contain activities many people share and that are considered part of life rather than part of a job, sports or dedicated activity. The list of typical ADL thus contains washing face, putting toothpaste on a toothbrush and brushing teeth or attaching the end of a zipper and zipping a jacket. ADL are typically very light activities
CBPA: Customized body-powered arm. We used this abbreviation to refer to the customized version of body-powered technology as laid out in the method section of our paper
COG: Center of gravity. With the center of gravity of a prosthetic arm for below elbow amputation located more distally, higher elbow torques result. Typing over a certain amount of time entails repetitive strokes. These may add to cause strain on arm, shoulder, neck and muscle pain
deg C: Degrees Celsius
g: Grams
ILT: Inherent technology limitation, such as limiting aspects of myoelectrode function (i.e., loss of function due to sweat, skin burn) that cannot be simply resolved by user modifications or user driven innovation
kg: Kilograms
MR: Manufacturer’
s restriction. A manufacturer can restrict the functionality of a device for safety reasons. An example is that a manufacturer can require the user to only operate an electronic prosthetic hand while it is covered by a water proof, manufacturer issued glove; MSL: Manual Skill Level. We used this subjective measure to rate the manual difficulty of single death scenes in this forensic medicine workplace evaluation
OB: Otto Bock is a German prosthetic component manufacturer
PDW: Physically demanding work with repetitive, strenuous, sweat driving or hazardous characteristics
TBI: Touch Bionics iLimb. We used this abbreviation to refer to the myoelectric device used for comparison against the CBPA. Its details and setup are described in the method section
VC: Voluntary closing mode of control for body-powered arms. Thereby, the terminal device rests in an open state and is closed, for the purpose of gripping or holding, by actuating the cable. It contains a spring mechanism that opens it again once the cable is relaxed again. During the actuation, the user feels the cable being tense and can react to variations in that feeling in real time. A typical device is the TRS Prehensor
VO: Voluntary opening mode of control for body-powered arms. Thereby, the terminal device rests in a closed state, pulled close by rubbers or springs. There are terminal devices where the user can add (or take away) rubbers or springs in order to modify the grip force. The device only opens once the cable is actuated. This type of device is particularly useful for holding or carrying items. Typical devices are Becker hands or Hosmer hooks
SHAP: Southampton Hand Assessment Procedure. A clinically validated hand function test
TRS: TRS is a company in Boulder, CO, USA that manufactures and sells high performance body powered prosthetic technology
UBEA: Unilateral below elbow amputee
USD: US-Dollars
Not contained in this list of abbreviations: abbreviations of authors’names

## Footnotes

1 WS, dominant sided right below elbow amputation 2008, board certified specialist in forensic medicine

2 detailed notes across the years on www.swisswuff.ch/tech

3 From Wikipedia: “Industrial and manual workers often wear durable canvas or cotton clothing that may be soiled during their work. Navy and light blue colors conceal potential sweat, dirt or grease on the worker’s clothing, helping him or her to appear cleaner.”

4 Swiss Insurance Law UVG article 11 contains that supportive measures [incl. prosthetic arms] must be simple and efficient [as to their intended purpose].

5 From [47]: “Health professionals offered little real help. Farmers in this study reported that health professionals assigned to assist them in their recovery were stymied because they lacked basic knowledge and understanding of farming.”

6
http://www.virtopsy.com

7 According to KHMI (Kreisschreiben über die Abgabe von Hilfsmitteln durch die Invalidenversicherung), paragraph 1036, Swiss laws provide that “instruction to use of the prosthesis is contained in the price at purchase” [215].

8 Internet query performed in September 2016
